# 

**Published:** 1918-04-06

**Authors:** 


					ItoDEXEn H MUL JM
/O 3C->?
The Modern Newspaper of Administrative
Medicine, and Institutional Life.
Ttlegraphic Address : OSPEDALE,  Telephone Nurgber; 27j4 QERRARjB.
No. 1661, Vol. LXIV. j[ ^Ipfturjday,
April 6th,
(J #PRufe ONfe PE^NV/

				

